# Moderate neutropenia with adjuvant CMF confers improved survival in early breast cancer

**DOI:** 10.1038/sj.bjc.6601366

**Published:** 2003-11-11

**Authors:** D A Cameron, C Massie, G Kerr, R C F Leonard

**Affiliations:** 1 Department of Oncology, Edinburgh University, Western General Hospital, Crewe Road South, Edinburgh EH4 2XU, UK

**Keywords:** adjuvant, CMF, early breast cancer, neutropenia, survival

## Abstract

Despite the extensive literature clearly demonstrating the survival benefit for adjuvant chemotherapy in women with operable breast cancer, there are few data confirming this in routine practice. Some studies have suggested that not all women gain to the same extent, with older women showing a smaller benefit and lower doses achieving poorer outcomes. We therefore reviewed the case notes of 750 women treated over a 15-year period at The Edinburgh Cancer Centre with the same intravenous CMF (cyclophosphamide, methotrexate and 5-fluorouracil) regimen, to identify patient- and treatment-related factors influencing outcome in routine practice.The actuarial 10-year survival for these women was 59.3%, with the anticipated poorer outcome for those with more involved ipsilateral axillary nodes, higher grade and ER-negative tumours. There was no evidence that a lower delivered dose intensity or older age at presentation resulted in a poorer survival. Of particular interest was the observation that 45% of patients who had grade 2/3 neutropenia had a 10% absolute survival advantage over those with no neutropenia (*P*<0.001). This strongly suggests that some degree of neutropenia has more influence on outcome than age or delivered dose intensity.

It is clear from the Early Breast Cancer Trialists' Collaborative Group overview analyses, and from many of the individual trials that have contributed to those meta-analyses, that adjuvant polychemotherapy improves the survival for women with early breast cancer (Early Breast Cancer Trialists Collaborative Group, 1992, 1998). Much debate has followed as to the optimum duration and schedule, and several studies have addressed these questions. At least one prospective study has shown that lower doses of cyclophosphamide, adriamycin and 5-fluorouracil are associated with a poorer outcome (Wood *et al*, 1994). Retrospective analysis of the pivotal Milan trial with classical CMF (cyclophosphamide, methotrexate and 5-fluorouracil) noted that those patients who received less than 85% of the planned dose did not fare as well as those who did and, more importantly, that those who received less than 65% of their planned dose had a survival no better than the untreated patients in the control arm (Bonadonna *et al*, 1995a). It was noted in that study that there was a strong tendency for the older patients to be undertreated, and in a subsequent study from the same group, looking at alternating CMF and adriamycin, it is clear that older women had a poorer outcome than younger women on the same regimen (Bonadonna *et al*, 1995b). Interestingly, in a recent audit of over 300 patients treated with that same adriamycin-CMF regimen in 11 centres in UK, Ireland and New Zealand, there was no difference in outcome for older patients (Cameron *et al*, 2002). These data, together with the observation from the meta-analysis that the benefit of adjuvant chemotherapy appears to diminish with increasing age, led us to hypothesise that it may be a failure to deliver drug dose and/or dose intensity in the older adjuvant breast cancer patient that results in its apparent reduced efficacy.

In contrast, there are clear retrospective data to suggest that patients who experience at least some degree of neutropenia while on their adjuvant chemotherapy may have an improved survival. The first study to report that neutropenia detected by a routine nadir full blood count predicted for a better outcome, utilised a regimen containing an oral fluoropyrimidine and could therefore be explained on the basis of variable absorption of the oral medication (Saarto *et al*, 1997). A more recent study looking at patients treated with adjuvant CMF regimens in Toronto also reported similar findings (Mayers *et al*, 2001).

We therefore additionally hypothesised that patients with evidence of neutropenia while on a totally intravenous CMF regimen would have a better survival than similar patients for whom there was no evidence of myelotoxicity.

## MATERIALS AND METHODS

Patients were identified within the departmental database as having been prescribed to receive six cycles of intravenous CMF (*vide infra* for doses) as adjuvant postoperative chemotherapy for a diagnosis of invasive breast cancer. Patients were to have started treatment between the years 1984 and 1998. Before 1984 there were very few patients, and they had been treated with an oral cyclophosphamide-based regime, the so-called ‘classical CMF’. The year 1998 was chosen as the cutoff in order to give a minimum follow-up period of 2 years. We identified 750 patients who met the criteria. The notes were then examined, and details of the dose and timing of each cycle of CMF were retrieved, along with any comments on toxicities, infections and the lowest recorded neutrophil count for each cycle.

Disease-free and overall survivals are measured from the date of first chemotherapy (or radiotherapy if that was given first), and are cause-specific except where stated.

### Treatment

All patients had to have been planned to be treated with six cycles of intravenous CMF on a 21- day cycle. In all, 559 women (75%) were given the standard regimen at the following doses: cyclophosphamide, 750 mg m^−2^; methotrexate, 50 mg m^−2^ and 5-fluorouracil, 600 mg m^−2^.

Of the women (including the majority of the patients over 60 years), 154 (21%) were given only 600 mg m^−2^ of cyclophosphamide. The remaining 37 patients had either some other planned dose or the planned dosage m^−2^ was not clearly defined (although the actual doses were). All other treatments received (surgical, radiotherapeutic and hormonal) were noted, but did not form a basis for including or excluding patients from the audit.

### Dose intensity

The usual policy for radiotherapy throughout this period was to administer it between either the first and second cycles in the earlier years, or between the third and fourth cycles of chemotherapy. Thus, patients scheduled for radiotherapy would automatically have a lower received dose intensity by virtue of this planned gap in chemotherapy administration. Therefore, analyses of dose intensity were made with and without an allowance for this gap. Since the standard cycle interval was 21 days, and there can be no delay after the final cycle, dose intensity was calculated for all patients receiving six cycles as


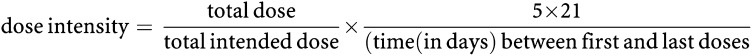


### Neutropenia

The grade of neutropenia was based on the lowest recorded neutrophil count for a patient between the day of first chemotherapy and 3 weeks after the final dose administered. The grading system used was that of the NCIC (so that grade 2 equates to a neutrophil count of between 1.0 and 1.5 × 10^9^ cells l^−1^ and grade 3 equates to a count of between 0.5 and 1 × 10^9^ cells l^−1^).

### Statistics

**Figure 1:**
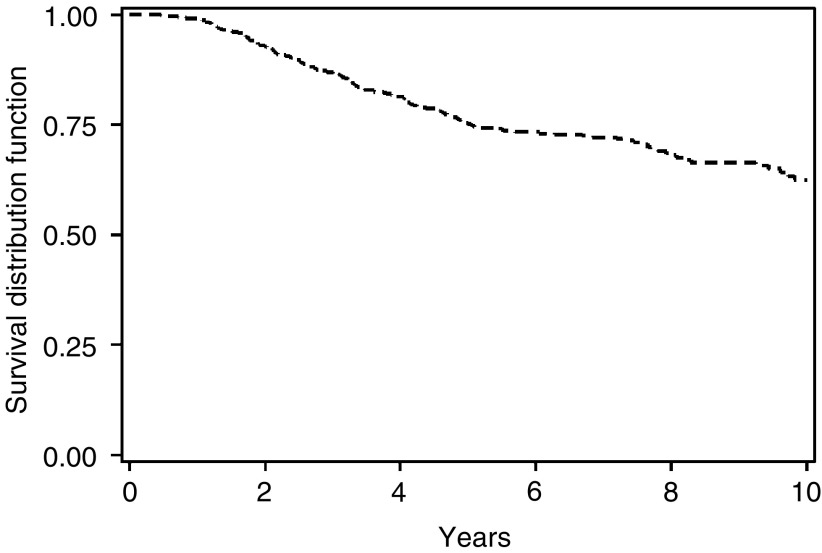
Breast-cancer-specific overall survival for all 750 patients treated with three-weekly i.v. CMF.

All statistical analyses and survival curves were produced using SAS Proprietary software (Copyright 1999–2001, SAS Institute, Cary, NC, USA). Survival data (including Figure 1

) are breast cancer specific unless otherwise stated. Proportional hazards analysis was performed for all patients who completed six courses of CMF chemotherapy. All patient-related, pathological and treatment-related variables were entered into the model and the analysis was performed by backward elimination.

## RESULTS

### Demographics and tumour details (see Table 1)

**Table 1:** Summary of patient and tumour characteristics by year of treatment

	**1984–1992**	**1993–1995**	**1996–1998**	**Total**
Number of patients	131	216	403	750
Median age (years)	43	46	50	47.9
Pathological size (mean)	26.5	22.3	22.5	23.1
% T1	28	32	39	35
% T2	64	60	55	58
% node negative	18	23	41	32
% 1–3 node positive	54	60	49	53
% 4–9 node positive	23	14	8	12
% grade 3	84	56	70	67
% ER positive/rich	35	56	51	50
% post menopausal	16	23	38	30
% over 60 years	3	7	17	12
% over 70 years	0	2	2	2
% mastectomy	33	44	40	40
5-year survival (%)	65.4	74.4	74.1	71.5

ER, Oestrogen receptor; XRT, Radiotherapy.

The majority of patients (83%) were treated after 1993, and were most commonly premenopausal (70%), stage II disease (70%), grade 3 cancers (67%) and/or with pathologically involved lymph nodes (68%). In all, 80% tumours were classified as ductal/no special type. ER status was known to be positive (>19 fmol mg^−1^ or histoscore >80) in 46%, poor (detectable but below the criteria for positive) in 23%, completely negative in 23% and unknown in 8%.

The median age overall was 47.9 (range 24–76) years, but increased significantly over time so that it was 43 years for patients treated between 1984 and 1992, 46 years for those treated between 1993 and 1995, and 50 years between 1996 and 1998.

### Treatment details

These have been summarised in Table 2

**Table 2:** Summary of patient, tumour and treatment characteristics by age (years) of patient

	**<40**	**40–49**	**50–59**	**60–69**	**70+**
Number of patients	149	304	194	89	14
Pathological size (mean) (mm)	23.0	23.6	21.6	24.4	29.2
% T1	33	32	41	38	29
% T2	61	59	54	55	64
% node negative	45	30	30	24	14
% 1–3 node positive	48	52	58	53	43
% 4–9 node positive	6	15	11	15	21
% grade 3	75	65	61	71	85
% ER positive/rich	38	54	55	43	50
% given tamoxifen	40	45	58	68	83
% post menopausal	3	6	51	100	100
% mastectomy	36	40	41	44	50
% mastectomy patients given radiation therapy	53	61	46	69	71
% completed six courses	93	93	94	92	79
% achieving 85% dose intensity	23	21	23	11	9
5-year survival (%)	65.5	78.9	78.9	70	68.3

ER, Oestrogen receptor; XRT, Radiotherapy.

. Of note is that for the 606 patients who received radiotherapy, it was intercalated in 84%, given before chemotherapy in 7% and given after chemotherapy in the remaining 9%. The majority (56%) had their radiotherapy between the third and fourth courses of chemotherapy.

Adjuvant hormonal therapy was known to have been given to 47% of patients. In 42% of patients tamoxifen was given, and 5% had ovarian ablative therapy (±tamoxifen). The vast majority of patients given endocrine therapy had some degree of ER expression, with only 24 patients being given tamoxifen for ER totally negative tumours. Among patients with ER-poor tumours, 37% were known to have received endocrine therapy, and 83% of those with ER-positive/rich tumours received further hormonal therapy.

### Chemotherapy delivery

A total of 53 (7%) patients failed to complete six cycles of chemotherapy. This was significantly more common in older women, with 8% of women aged 60–69 years and 21% of women over 70 years failing to complete their planned therapy (*χ*^2^ for trend=5.7, *P*<0.02).

Treatment delays were surprisingly common. Overall, we noted that 76.6% of patients and 37.5 % of courses had a delay of at least 1 day. Delays for reasons of toxicity occurred in 54% of patients, with a delay of at least a week occurring in 44% of patients. There was however no evidence that delays were more common in older patients.

### Documented toxicity

Grade 4 neutropenia (neutrophils <0.5 × 10^9^ l^−1^) was documented in 76 (10%) patients, including two of 14 of those over 70 years , and 101 of 4255 (2%) of courses. There is an apparent trend for a lower incidence of neutropenia in later courses, with, for example, 4% following the first course, 3% after the fourth and only 1% in the final course (*χ*^2^ for trend across all six cycles=16, *P*<0.001). It is clear however that in the absence of toxicity, very few patients had a routine full blood count after cycle 6. Therefore, the data from that final cycle could be an underestimate, but this would not in any case have altered treatment delivery. Of the 76 patients with at least one documented neutrophil count below 0.5 × 10^9^ l^−1^, all but two completed all six cycles (neither of whom was over 70 years old), one of whom had three episodes of grade 4 neutropenia before the final cycle was omitted. All but two patients, however, had either a dose reduction (47%) or a treatment delay.

A highest recorded grade of neutropenia of 2 or 3 was documented in 133 (17%) and 208 (28%) patients and 5% and 7% of courses, respectively. Patients with grade 2/3 neutropenia were significantly younger (mean age 48 years) than those with grade 4 neutropenia (mean age 52 years, *P*<0.002), and overall there was a significant trend for higher grades of neutropenia in older patients (*χ*^2^ for trend=6.87, *P*<0.01).

### Patient outcome

**Figure 2:**
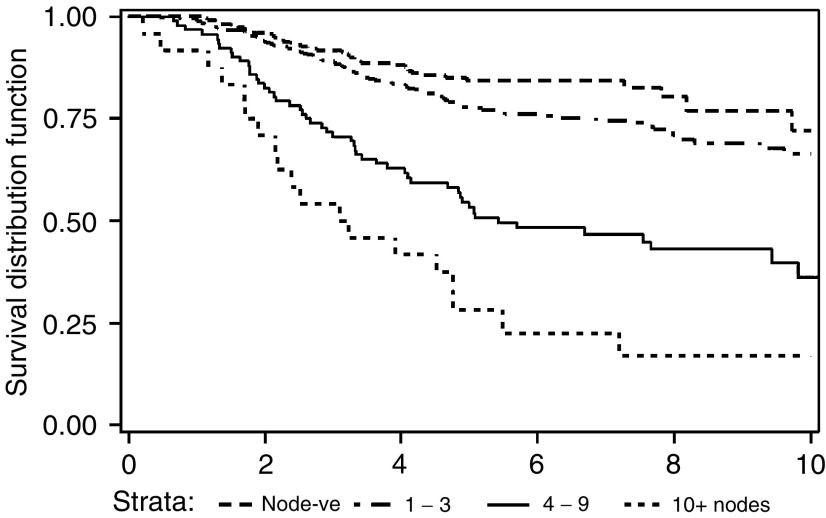
Breast-cancer-specific overall survival by number of involved axillary nodes (*P*<0.0001).

Three patients were lost to follow-up in the first 2 years, leaving a median follow-up of 4 years for the remaining patients. In all, 17 patients died a nonbreast cancer death, including two that were probably treatment related (one at home midcycle, and the other following presumed neutropenic sepsis). A total of 260 patients have documented relapse of breast cancer, of whom 202 have died of breast cancer. Actuarial all-cause overall survival was 73.2, 59.3 and 50.3 % at 5, 10 and 15 years, respectively. Breast-cancer-specific survival rates were 74.6, 62.2 and 52.7%, respectively (see Figure 1). The strongest predictor of overall survival was the number of involved axillary lymph nodes, *P*<0.0001 (see Figure 2

). As might be expected, ER-rich tumours were associated with the best overall survival of 81% at 5 years as compared with 64% for ER-poor and 70% for ER-negative tumours.

There was no evidence of any difference in survival between the three cohorts (see Table 1). The 5-year survival by age is shown in Table 2, where it can be seen that women aged between 40 and 49 years had the best survival (*P*=0.0019). Women over 60 years may appear to have a poorer survival than those aged between 40 and 59 years, but it is also apparent from Table 2 that they have a higher proportion of tumours with poorer prognostic factors, for example, within the four categories of nodal involvement (negative, 1–3, 4–9 and 10+); there is no evidence of a poorer disease-free survival for any particular age group.

Figure 3

**Figure 3:**
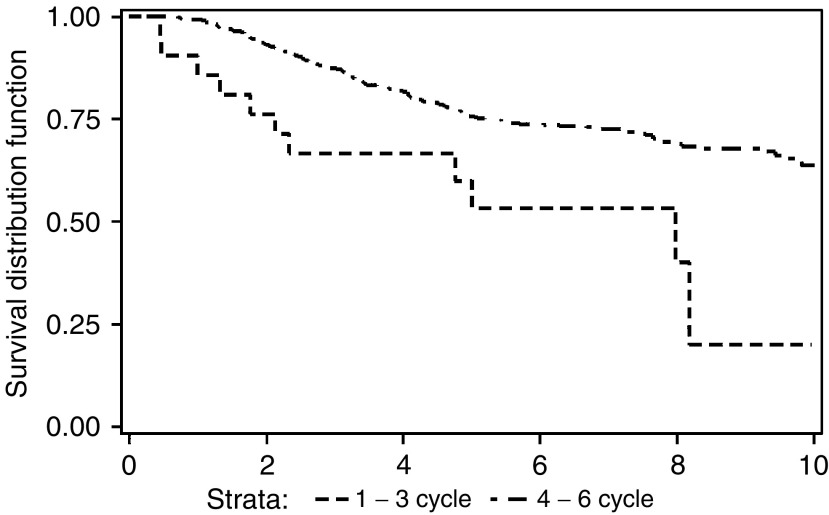
Breast-cancer-specific overall survival by number of courses of CMF administered (*P*=0.009).

shows that the small number of patients (23) who failed to receive more than three courses had a significantly poorer survival (*P*=0.009). However, there was no evidence that lower received dose intensities, in the 687 patients completing all six courses, were associated with any differences in survival (see Figure 4

**Figure 4:**
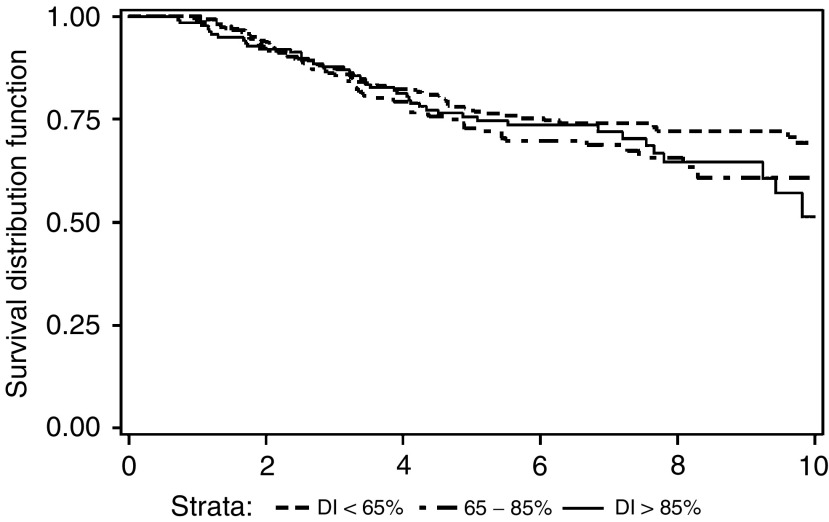
Breast-cancer-specific overall survival by average dose intensity for patients completing six courses of CMF chemotherapy (*P*=NS).

). There was no evidence that the actual timing of the radiotherapy had any effect on outcome (data not shown), although it should be noted that only 43 and 53 patients, respectively, received radiotherapy before and after all their chemotherapy (with 5-year survivals of 69 and 71%, respectively).

Figure 5

**Figure 5:**
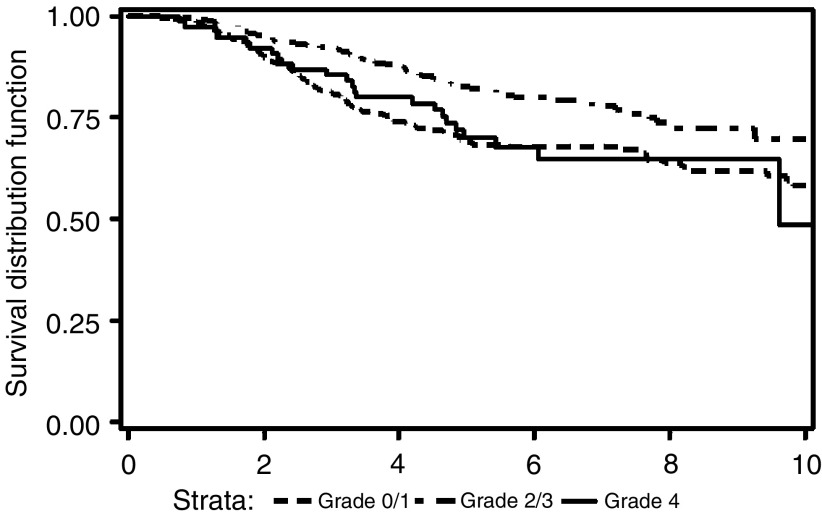
Breast-cancer-specific overall survival by maximum recorded grade of neutropenia (*P*<0.001 for grade 2 and 3 combined *vs* grades 0, 1, and 4 combined).

shows the perhaps surprising result that there is an absolute improvement in overall survival of over 10% for those women whose maximum recorded level of neutropenia was grade 2 or 3, as compared with those women who experienced grades 0, 1 or 4 neutropenia. This difference is seen across all patient and tumour categories, although in some of the smaller groups (the earliest cohort, grade 1 and 2 tumours, and ER-rich tumours) the observed difference is not statistically significant (see Table 3

**Table 3:** Details of survival for patients with grade 2/3 neutropenia

**5-year cause-specific survival**	**Grades 0, 1, and 4**	**Grades 2 or 3**	***P-*value**
Number of patients	397	353	
Overall	68%	82%	<0.0001
1984–1992	64%	71%	NS
1993–1995	66%	82%	0.04
1996–1998	72%	86%	0.0027
Age <50 years	72%	82%	0.017
Age >50 years	61%	81%	0.0006
ER negative	59%	83%	0.0008
ER poor	58%	75%	0.008
ER rich	79%	85%	NS
Grades 1 and 2	79%	91%	NS
Grade 3	66%	76%	0.011

NS, nonsignificant; ER, Oestrogen receptor; XRT, Radiotherapy.

). Furthermore, if the comparison is made between, on the one hand, those patients with grade 0 or 1 neutropenia, and on the other hand those experiencing grade 2 or higher, the latter continue to have a statistically significant better survival (*P*=0.001), also seen in every subgroup (although no longer reaching significance in women with ER-poor tumours) (data not shown).

In multivariate analysis, the degree of neutropenia remains an independent predictor of a poorer outcome along with the number of involved axillary nodes, a low ER of the tumour, and the patient having had a mastectomy (see Table 4

**Table 4:** Multivariate analysis for relapse-free and cause-specific overall survival at 5 years

**Variable**	**Overall survival**
Number of lymph nodes	*P*<0.0001
Grade 2 or 3 neutropenia	*P*=0.0377
ER poor/negative	*P*<0.0001
XRT to supraclavicular fossa	*P*=0.0169
Mastectomy	*P*=0.004
XRT to primary site	*P*=0.041

Proportional-hazard analysis was performed for all patients who completed six courses of CMF chemotherapy. All patient-related, pathological and treatment-related variables, including pathological tumour size, were entered into the model and the analysis was performed by backward elimination. Only 497 patients were included in the analysis, due to incomplete information on some variables; in particular, grade was not available for 136 patients and ER status for 60 patients. Those variables having a significant effect on cause-specific survival are shown in the table; ER, Oestrogen receptor; XRT, Radiotherapy.

). It is also clear that having a mastectomy is an indicator of poorer outcome, probably because of its relationship with larger tumour size: there was a mean pathological size of 27.6 mm as compared to the statistically significantly smaller mean of 20.2 mm for the women having breast conservation (*P*<0.0001).

## DISCUSSION

This retrospective analysis was carried out in order to see if older women fared worse with adjuvant intravenous CMF as a consequence of more toxicity and/or lower doses and dose intensity. Clinicians at our institute had often reduced the dose of cyclophosphamide, as commented on previously, in anticipation of higher toxicity in older women. The data presented here, however, suggest that despite a moderate increase in toxicity, there is no evidence of a lower dose intensity and poorer outcome for older women

What is very clear, however, is that in our study, those women, irrespective of their age, who had moderate neutropenia had a substantially better survival, with a similar level of gain being seen within each prognostic group (see Table 3). In two small groups (the first cohort and those with grade 1 and 2 tumours), the difference is not statistically significant, and in women with ER-positive tumours, any difference observed is similarly not statistically significant. However, similar trends are also seen for disease-free survival, and in no subgroup is the outcome for women without neutropenia better than for those with a moderate degree. Thus even though some observed differences do not reach statistical significance, they remain consistent with the observation that modest neutropenia accords a survival advantage in the adjuvant use of CMF. It is not entirely clear as to why the 10% of patients who experienced the most severe neutropenia had a similar outcome to those women who had no neutropenia, but one important difference may be that they were on average older. However, as commented on above, the improved survival persists if they are included in the moderate neutropenia group. Thus, these data are consistent with the hypothesis that a lack of neutropenia suggests underdosing, and similar findings have been reported by others.

Comparable data for a CMF-based regimen come from Canada, where even in a multivariate analysis, experiencing grade 3 or 4 neutropenia correlated with better survival (Mayers *et al*, 2001), in a similar manner to the advantage seen in this study for those women experiencing grade 2 or 3 neutropenia. Three other factors were also associated in univariate analyses with an improved outcome, namely inclusion in a clinical trial, delivery of a dose intensity above the median and/or the use of the classical CMF regimen. However, the magnitude of the benefit associated with these factors was considerably smaller than that seen in women experiencing neutropenia, and none of these factors were significant in the multivariate analysis. In another, smaller study analysing the outcome of 211 women with early breast cancer administered an adjuvant regimen consisting of bolus doxorubicin and cyclophosphamide and an oral analogue of %-FU (ftorafur), Saarto *et al* (1997) reported a significant correlation between leucocyte nadir and both disease-free and overall survival. They also reported that any relationship between the dose intensity of doxorubicin and cyclophosphamide and outcome was only significant when patients stopped chemotherapy early, whereas that with the nadir leucocyte count persisted in a multivariate analysis.

One criticism of this study relates to the use of a three-weekly i.v. CMF regimen. We are in the process of comparing these data with data from another UK unit with 20 years' experience of using the ‘classical’ or Bonadonna CMF, but the results are not yet available. It is interesting, however, to note how the outcome for the women in our study compares very favourably with that recently reported by the IBCSG and GBSG, in their combined analysis of three and six cycles of adjuvant ‘classical’ CMF (Colleoni *et al*, 2002), with almost identical 5- and 10-year survivals. There are of course differences in demographics and risk factors (patients in our study were on average older, less likely to have four positive node tumours, but equally less likely to have ER-positive tumours).

Thus our data, as well as two other retrospective studies (Saarto *et al*, 1997; Mayers *et al*, 2001), strongly suggest that it is the achievement of neutropenia that could be fundamental to delivering optimal adjuvant chemotherapy.

However, this is not a justification for high-dose chemotherapy, for which preliminary data from a number of adjuvant studies have failed to show any clear advantage for increasing the dose of adjuvant chemotherapy above which is tolerable without growth factor or bone marrow stem cell support. In contrast, a small number of other studies have shown that a planned reduction in dose is detrimental, such as the CALGB study (Wood *et al*, 1994), retrospective analyses from the Institute of Cancer in Milan (Bonadonna *et al*, 1995a) as well as a study randomising patients to different doses of epirubicin (Piccart *et al*, 2001).

How can we reconcile these data? It is clear from the small study from Newcastle, UK, looking at the variable pharmacokinetics of adjuvant CMF in breast cancer, that there is significant interpatient variability (Batey *et al*, 2002). We suggest that the current trend for prescribing chemotherapy using an estimated body surface area is insufficiently precise to ensure that all patients receive a sufficient dose, and that our data support a pharmacodynamic definition of adequacy being that which induces modest neutropenia. If there was a simple way in which this could be ensured for every patient, then our data, as well as those of others, would suggest that there would be an overall improvement in survival, possibly as large as that sought but not yet found, in studies of myeloablative chemotherapy. Support for this hypothesis was apparent in a study recently reported by the Swedish group. In their randomised trial, they demonstrated that high-dose chemotherapy appeared to be inferior to an anthracycline-based regime with dose escalation according to toxicity (Bergh *et al*, 2000).

Although it remains a conundrum as to why the plateau of dose–response in adjuvant chemotherapy appears around the dose that individually delivers modest neutropenia, the data we and others report strongly call for studies to test the hypothesis that adjuvant chemotherapy in early breast cancer has to cause myelosuppression to be optimal.
